# The Use of a Quasi-Experimental Study on the Mortality Effect of a Heat Wave Warning System in Korea

**DOI:** 10.3390/ijerph16122245

**Published:** 2019-06-25

**Authors:** Seulkee Heo, Amruta Nori-Sarma, Kwonsang Lee, Tarik Benmarhnia, Francesca Dominici, Michelle L. Bell

**Affiliations:** 1 School of Forestry and Environmental Studies, Yale University, New Haven, CT 06520, USA; amrutasri.nori-sarma@yale.edu (A.N.-S.); michelle.bell@yale.edu (M.L.B.); 2 Harvard T. H. Chan School of Public Health, Harvard University, Boston, MA 02115, USA; kwonsanglee@hsph.harvard.edu (K.L.); fdominic@hsph.harvard.edu (F.D.); 3 Department of Family Medicine and Public Health and Scripps Institute of Oceanography, University of California San Diego, La Jolla, CA 92093, USA; tbenmarhnia@ucsd.edu

**Keywords:** hot temperature, mortality, heat waves, extreme heat, climate change, quasi-experiment, vulnerability, adaptation, heat action plans

## Abstract

Many cities and countries have implemented heat wave warning systems to combat the health effects of extreme heat. Little is known about whether these systems actually reduce heat-related morbidity and mortality. We examined the effectiveness of heat wave alerts and health plans in reducing the mortality risk of heat waves in Korea by utilizing the discrepancy between the alerts and the monitored temperature. A difference-in-differences analysis combined with propensity score weighting was used. Mortality, weather monitoring, and heat wave alert announcement data were collected for 7 major cities during 2009–2014. Results showed evidence of risk reduction among people aged 19–64 without education (−0.144 deaths/1,000,000 people, 95% CI: −0.227, −0.061) and children aged 0–19 (−0.555 deaths/1,000,000 people, 95% CI: −0.993, −0.117). Decreased cardiovascular and respiratory mortality was found in several subgroups including single persons, widowed people, blue-collar workers, people with no education or the highest level of education (university or higher). No evidence was found for decreased all-cause mortality in the population (1.687 deaths/1,000,000 people per day; 95% CI: 1.118, 2.255). In conclusion, heat wave alerts may reduce mortality for several causes and subpopulations of age and socio-economic status. Further work needs to examine the pathways through which the alerts impact subpopulations differently.

## 1. Introduction

Climate change has led to an increase in annual average temperatures across the globe, with 2016 being the warmest year on record (followed by 2015, 2017, and 2018) [1,2]. State-of-the-science climate models indicate that under a variety of scenarios, this warming trend will continue in the coming decades. For example, according to the IPCC (Intergovernmental Panel on Climate Change) Fifth Assessment Report (AR5), the increase of global mean surface temperature is likely to be 2.6–4.8 under RCP8.5 (the highest CO_2_ emission scenario) by the end of the 21st century (2081–2100) compared to 1986–2005, which will certainly increase the frequency and duration of extreme heat events [3]. In Korea, the increase in annual average temperatures was estimated to be up to 5.3 °C and the annual extreme heat wave duration was projected to increase by 6.1 days in the late 21st century compared to the late 1990s according to the most severe greenhouse gas emission scenario [4]. Climate change poses extensive direct and indirect threats to public health; one of the primary effects of climate change is the health burden of high temperature and heat waves on mortality and morbidity [5].

Numerous studies have reported significant impacts of high temperature on mortality and hospitalizations in Korean populations [6,7,8]. Additionally, the impact of heat waves on the increase in mortality has been examined under different heat wave definitions based on the application of heat indices and the frequency and duration of heat waves [9,10,11]. Studies have shown that vulnerable groups for mortality due to short-term exposure to high temperature include people living in urban cities without adequate air conditioning, the elderly especially those living alone, children, people in poverty, and people with underlying health conditions that are exacerbated by heat [12,13,14,15,16].

Many countries, including Korea, have been developing and applying policies for preventing health burdens from extreme weather and improving resilience of societies to heat-related public health damages. Evidence-based policy making is crucial for continuous successful performance of these policies, as well as the development of additional health-protective policies in other countries that are likely to experience the effects of extreme weather, especially in the context of climate change.

In response to the growing concern of impacts of heat waves on public health, in 2008 Korea launched national heat wave health plans [17] that are activated by the heat wave alert system administered by the Korea Meteorological Administration. The heat wave health plans aim to warn the public and health authorities of the impacts of heat waves, and to mitigate the associated negative health consequences [18]. The plans have been developed with growing cooperation among related ministries and municipalities [17]. Heat wave alerts are issued by forecasts for regions where daily maximum temperature is expected to be 33 °C or above for 2 or more consecutive days based on the alert system [19]. One common threshold temperature of 33 °C is used for every region.

When a heat wave alert is announced, the heat wave department of the local municipalities immediately receives a warning bulletin from the Korean Meteorological Administration. While any person could be impacted by a heat wave, some implemented heat wave preventive actions are applied to groups that are anticipated to be particularly vulnerable, including the elderly (age 65 and more), disabled people, people who live alone, outdoor workers, and homeless peoples. The heat wave health plans incorporate various measures to be undertaken by the local government, such as opening shade shelters at night and on weekends/holidays and dispatching health care providers to the target vulnerable subpopulations. The Korean Ministry of Employment and Labor operates educational programs on heat-related health risks for construction and shipbuilding sites during warm seasons. When an alert is announced, ministries instruct local officials to operate break times (2–5 pm) at work sites for the elderly (age 65+) and outdoor workers (e.g., farmers, manual laborers, and soldiers), and at schools for young students. The Korea Center for Disease Control and Prevention operates ‘surveillance systems for heat-related illnesses’ during the warm season to monitor morbidity due to heat-related illnesses across the country. Further, it is instructed to supply heat-related first aid items (e.g., ice pack, saline solution) to local ambulances. While implementation of preventive measures by local municipalities can vary among regions, the national heat wave health plans have continuously grown by encouraging municipalities to develop and operate their own plans optimized for local situations. 

While the significance of the relationship between heat waves and mortality has been demonstrated and national-scale plans have been executed many countries and cities [20], little is known about whether the implementation of heat wave alert system and concurrently executed heat wave health plans (here on, referred to jointly as ‘the heat wave warning system’) produces a positive effect on reducing the mortality associated with heat waves. Evaluations of the heat wave warning system will inform evidence-based interventions aimed at reducing heat-related health impacts. We aimed to evaluate the effectiveness of the heat wave warning system to prevent the impact of heat waves on mortality in Korean populations for 2009–2014, utilizing the accuracy of the heat wave alert system in detecting actual heat wave days and the discrepancy between the early heat wave alerts and the actually monitored temperature.

## 2. Materials and Methods 

### 2.1. Data

The study period was the warm seasons (June to September) for 2009–2014. Although the heat wave warning system was implemented in 2008, this year was excluded from the study period because of incompleteness of data in the warm season. We targeted 7 cities in Korea (Seoul, Busan, Incheon, Gwangju, Ulsan, Daejeon, and Daegu). The 7 cities are the most populated metropolitan cities in Korea and they have been considered in several previous studies [10]. The spatial unit was based on ‘Gu’, which is equivalent to the US spatial unit ‘borough’. We obtained mortality data, weather data including monitoring and heat wave alert announcement data, and air quality monitoring data for each Gu in the study cities for the period of 2009–2014. 

The mortality data included information on age, sex, day of death, cause of death, education attainment, job status, and marital status of individual deaths reported every year. Cause of death was categorized based on the ICD (International Statistical Classification of Diseases and Related Health Problems) 10th codes. Using mortality data, we calculated daily all-cause death counts by age groups (0–19, 20–64, 65+, 75+ years), sex, occupation (white-collar, blue-collar, unemployment), marital status (single, married, widowed, divorced), and education level (none, elementary, 7–12th grade, university or more), as well as cardiovascular and respiratory mortality of all ages. For education attainment, we conducted analysis for adults to avoid misclassification of children (who are still in elementary education years) into the group of people with completed education of high school or less. Similarly, for occupation and marital status, we limited the analysis to adults. For analysis focusing on job status, marital status, and education level, mortality was parsed into age groups 19–64, 65+, and 75+ years (as age 19 is the age when individuals commence university education in Korea).

Heat wave alert announcement data and weather monitoring data for 2009–2014 were obtained from the Korea Meteorological Administration. The heat wave alert data were available at the city and the prefecture level. The dataset included information on the date and time when a heat wave alert was issued for a given region. Using the heat wave alert announcement data, we created an indicator variable of heat wave alert forecast (i.e., 1: heat wave, 0: non-heat wave) for each day and each study city. Note that this indicates when a heat wave was anticipated to occur, not when a heat wave actually occurred. We merged this data with the mortality data, individually for each Gu. Weather monitoring data included daily mean and maximum temperature (°C), daily mean relative humidity (%), and daily mean wind speed (m/s). There were no missing values for daily mean temperature or relative humidity, whereas the percentage of missing values for daily mean wind speed was 0.2% (95 days out of 50,027 days). To adjust the effect of air pollution on mortality risk during warm seasons, we obtained the 24-hour average concentrations of particles 10 μm or smaller (PM_10_) and ozone (O_3_) from the Korean National Institute of Environmental Research. Data for PM_2.5_, which are technically verified by the institute, were not available for the study regions and the study period. The percentage of missing values of weather variables for the study period was 0%. The percentage of missing values for PM_10_ or O_3_ for the study period was 1.7%. 

### 2.2. Statistical Analysis

#### 2.2.1. Approach of Difference-In-Differences Model 

Heat wave alerts are forecasted with a great amount of accuracy, but they are not perfect. The detection rate of the heat wave alert system for true heat wave or non-heat wave days was 94.5% for the study period and the percentage of false negative and false positive heat wave days together was 5.5%. This imperfect feature of forecasting provides an interesting natural experiment design that allows us to estimate the effectiveness of the heat wave warning system. Each day can be classified with two variables: (1) whether a day is forecasted as a heat wave day and (2) whether a day is an actual heat wave day (i.e. temperature ≥33 °C for 2 or more consecutive days). This classification generates four different categories: (1) true heat wave days that were correctly identified as such by the alert system, (2) true non-heat wave days that were correctly identified by the alert system (i.e., identified as non-heat wave days), (3) false positive heat wave days (i.e., days identified by the alert system as heat wave days, but that were actually not heat wave days based on the monitoring data), and (4) false negative heat wave days (i.e., days that were not identified as heat wave days by the alert system, but that were actually heat wave days based on the monitoring data). These four types of days are described in Table 1. 

To estimate the effectiveness of the heat wave warning system, the Difference-in-Differences (DD) approach can be considered. A DD method is a quasi-experimental research design that attempts to estimate unbiased effects in non-randomized settings, to aid evidence-based policy making [21]. In standard DD settings, there are treatment and comparison groups with two time points, before and after implementing an intervention [22]. We call these two time points Time 1 and Time 2. Both treatment and comparison groups are not affected by an intervention at Time 1 since an intervention is not implemented yet. However, only the treatment group is affected by the intervention at Time 2. The outcome difference within the comparison group between Time 1 and Time 2 represents the time trend in the outcome. The outcome difference within the treatment group between Time 1 and Time 2 is the sum of the time trend and the effect of the intervention. If we assume that this time trend is the same, the effect of the intervention on the outcome can be estimated. The same time trend assumption is often called “parallel trend assumption”. Under this parallel trend assumption, the difference in the outcome difference between the treatment and comparison groups represents the genuine treatment effect of the intervention with the adjustment of the time trend. Unlike the standard DD settings, there are no specific time points in our study design. Instead, a group of days without the heat wave alert is considered as “Time 1”, and a group of days with the alert is considered as “Time 2”. Actual heat wave and non-heat wave days are considered as the treatment and comparison groups, respectively. The outcome difference between Time 1 and Time 2 during the non-heat wave days represents the change in non-heat-related mortality (i.e., the time trend in standard DD designs). 

**Figure 1:**
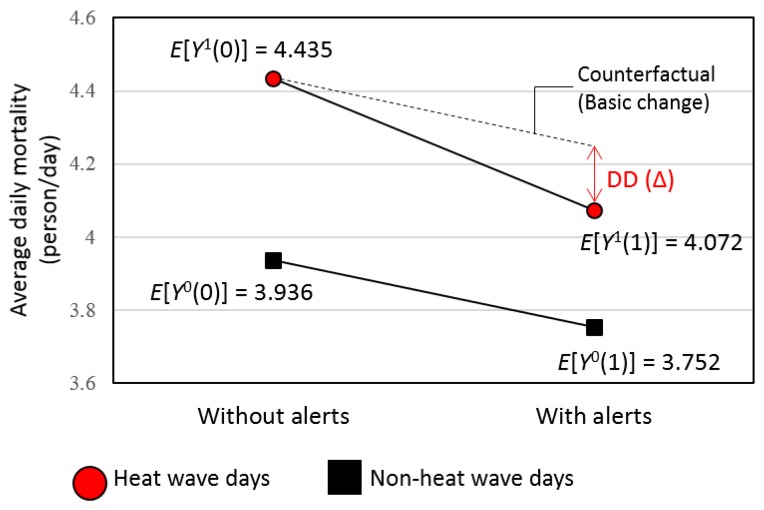
Average all-cause mortality (person) per day for the heat wave categories (2009–2014). Notes. DD: difference in difference, $E\left[Y^{0}\left(1\right)\right]$: mortality in false positive heat wave days, $E\left[Y^{1}\left(1\right)\right]$: mortality in true heat wave days, $E\left[Y^{0}\left(0\right)\right]$: mortality in true non-heat wave days, $E\left[Y^{1}\left(0\right)\right]$: mortality in false negative heat wave days. The noted DD is the estimated effectiveness of the heat wave alerts adjusted for the counterfactual mortality difference for non-heat wave days (comparison group) with and without heat wave alerts.

To illustrate our DD design, we defined the potential outcome $Y^{H}\left(i,\;A\right)$, where H indicates whether day *i* is a true heat wave day or not and A indicates whether day *i* is alerted by the heat wave alert system. For example, $Y^{0}\left(i,\;1\right)$ denotes the outcome that would be observed if day *i* is not a true heat wave day (based on monitoring data) but is forecasted as a heat wave day. To describe the average outcome for each type of heat wave days, we dropped the day argument *i* and use the expectation *E*[*Y^H^*(*A*)] with slight abuse of notation. Table 1 shows these expectations. The outcome difference *E*[*Y*^0^(1) − *Y*^0^(0)] between false positive and true non-heat wave days represents the change in mortality not related to heat. This is the time trend in the outcome for the comparison group in standard DD designs. In comparison, the outcome difference between true heat wave and false negative days (i.e., *E*[*Y*^1^(1) − *Y* ^1^(0)]) combines the effect of announcement with the effectiveness of interventions regarding health risk, that is, the change in sum of heat-related and non-heat-related mortality. Under the same parallel trend assumption, the effectiveness of interventions can be separated and estimated by computing difference in the two differences, *E*[*Y*^1^(1) − *Y*^1^(0)] and *E*[*Y*^0^(1) − *Y*^0^(0)]. This DD estimate was shown in the right lower cell of the table and Figure 1. 

If heat wave warning system is completely ineffective in preventing the health risk of heat waves, the DD estimate would be zero or larger. In the cases where the warning system itself does not contribute to the decreases in mortality during a heat wave, the DD estimates will be positive values indicating that the increase in mortality among groups may be attributable to the health effects of heat waves. If we expect that warning system has a protective effect, we anticipate that the DD estimate would be negative. The potential effectiveness of heat wave warnings is measured as a widening of the gap between the groups with intervention including the alert announcements and health protective steps and groups without the intervention (with this widening indicating that alerts are actually effective on preventing the health risk). We examined the degree to which the heat wave warnings reduce mortality associated with heat waves. Further, we stratified the data and assessed the effectiveness of the heat wave warnings, for individual-level vulnerability factors such as age, educational attainment, marital status, and occupation. 

Selection bias may occur if the heat wave alert system has different accuracy levels between announcing heat waves and non-heat waves. This may violate the baseline assumption of the same risk difference in the two groups that are affected and unaffected by the heat wave alerts in the absence of the alerts. Another way in which selection bias could occur is if the heat wave alert system selectively determines some days as being heat wave days. Days with extremely high temperatures will more likely to be determined as heat wave days and days with extremely low temperatures will more likely to be determined as non-heat wave days by the alert system. These two groups themselves would differ by the variation of temperature and therefore by the effect of temperature on mortality. To minimize these potential biases and obtain reliable estimates of the effect of interventions on mortality, we selected eligible days for the risk estimation as the days for which daily mean temperature were between 26.0 to 30.5 °C. This interval was selected by comparing the proportions of each heat wave category among different interval sets (Supplementary Figure S1). For example, if the intervals included days for which daily mean temperature was below 23.5, the proportion of true non-heat wave days was 100%. Likewise, for the days with daily mean temperature >30.5 °C, the proportion of true heat wave days was 100%. This approach stabilized the calculated propensity score weights and provided a further unbiased estimation of the effectiveness of the intervention on mortality associated with heat waves. 

#### 2.2.2. Propensity Score Weighting 

To minimize the potential selection biases and adjust for the effect of measured potential confounders (e.g., air pollution) on mortality, we also applied the multiple group propensity score weighting method to DD models [23]. The propensity score weighting method shows great performance for estimating unbiased effects of the intervention [24]. Within the context of the heat wave warnings on a given day, the propensity score is the probability that the day will be assigned as heat wave day by the alert system based on observed characteristics of covariates; the score is a number between 0 and 1 indicating the influence of all the observed covariates on the likelihood of being assigned a heat wave day by the alert system. Multinomial logistic regression models were applied to estimating propensity scores of each of the four groups in Table 1 (i.e., true heat wave days, true non-heat wave days, false positive heat wave days, and false negative heat wave days) in order for them to reflect similarity defined by some confounder and to estimate the causal effect in the entire days through the study period. 

In estimating propensity scores, the selected covariates are assumed to be independent baseline variables not affected by the assignment of heat wave alert itself. This is because post-intervention characteristics might have been affected by the alert, and using such variables in calculating propensity scores would bias the results of the effectiveness of the intervention [25]. Previous studies additionally suggested that including covariates unrelated to the assignment of intervention but related to the outcome (i.e., mortality) in the propensity score calculation can decrease the bias and the variance of the effect of intervention, as compared with estimates that omit such covariates [26,27]. Thus, in this study, we carefully identified covariates for calculating propensity scores by considering common covariates that have been shown previously to be important confounders of the relationship between short-term exposure to high ambient temperature and mortality [28,29]. We employed regression models using a set of those covariates to identify whether the baseline of those covariates is potentially correlated with the assignment of the heat wave announcement, and thereby acts as a confounder for the relationship between heat waves and mortality. After conducting multiple multinomial logistic regression analyses combining different sets of covariates as independent variables for the heat wave alerts assignment as the dependent variable and checking the model diagnosis (i.e., VIF, AIC), the following variables were identified to be incorporated as covariates in calculating propensity scores of each day in the four groups of heat wave types: daily mean humidity, wind speed, the average of O_3_ concentration through previous 3 days (lag 1–3), the average of PM_10_ concentration through previous 3 days (lag 1–3), year, day of the week, the difference between the same day mean temperature and the mean temperature a day before, an indicator variable for cities, the number of all ages population, and the proportion of elderly population (age 65+). We note that it is important to incorporate temperature or air pollution that are not from the same day (i.e., lag 0) for calculating propensity scores for each day because the variables on the same day might represent post-intervention characteristics. By balancing the sample days on these covariates, we can estimate the average changes in mortality among all sample days through the heat wave alerts, not through the different conditions of confounders among them [30]. 

The constructed weights from the propensity scores were subsequently applied to the weighted regression model in which the mortality rate (deaths per 10^6^ persons) Y, the outcome of this study, was expressed as a function of the indicator variable of heat wave alert announcement (*HA*) (i.e., intervention), the indicator variable of heat wave based on monitoring data (*HM*), and the interaction term between these two variables for each city:

(1) $$Y=\alpha +\beta_{f}HA+\;\beta_{m}HM+\;\beta_{fm}HA\times HM+\epsilon ,$$

where the estimate of *β_fm_* is taken as the DD estimate (Δ in Table 1), which is equivalent to the DD estimate from a nonparametric approach using stabilized weights. Technically, this weighted regression minimizes the weighted sum of squares. 

It should be noted that the DD positive DD estimates do not necessarily imply that the warning system contributes to increases in mortality. The DD estimates based on the proposed method in this study can be positive even with the reduced mortality mainly for two reasons: sensitivity to weight truncation and violation of the parallel trend assumption. The proposed weighted regression model (1) produces the valid DD estimate, but there can be a problem of extreme weights that can unduly influence results and provide high variance of the estimate [31]. To remedy this problem, we truncated the extreme weights from the analyses [32]. Excluding too many weights can result in biased estimates, since the target population may be distorted due to truncation. Therefore, it is important to find the optimal cut-off values to remove extreme weights while minimizing the impact of truncation on the target population. In preliminary results, we compared truncating the weights at different cut-off values (e.g., 99, 99.5, 99.9 percentiles) and selected the 99.5 percentile as truncations below that cut-off resulted more than 6% loss of false negative heat wave days from the eligible days for analysis. As a result, we selected the days whose calculated weights were ≤ 99.5 percentile of the weights and our central point estimates and standard errors for the DD were computed from the above model (1) with the constructed weights after truncation. To examine the sensitivity of the effects of the heat wave warning system on mortality change, we used propensity score weights under the 99th percentile of the weights in sensitivity analyses. The violation of the parallel trend assumption is further discussed in the discussion section.

The other sensitivity analyses included examinations for trend in effectiveness of the heat wave warning system over time after the implementation (e.g., 2009–2011 vs. 2012–2014) and for more age subgroups among children (0–4 and 5–19 age groups). 

#### 2.2.3. Subgroup Analysis 

We stratified the DD model for all-cause mortality for subgroups of age (0–19, 20–64, 65+, 75+), education attainment (none, elementary, 7–12th grade, university or more), marital status (single, married, widowed, divorced), and job status. As the definition of elderly population varies among studies [33], we examined both 65+ and 75+ age groups. Job status was categorized into white-collar, blue-collar, and unemployment in relation to outdoor heat exposure. White-collar workers included the executive, administrative, and managerial occupations, expert, engineer, and office workers. Blue-collar occupations included workers whose labor is related to service industry, sales, agriculture, fishing industry, technician, machine assembly, and labor work. Unemployment coding from the raw mortality data included the status of unemployment, as well as students and housewives. For education attainment, marital status, and job status, stratified analyses were applied to age subgroup of 19–64, 65+, and 75+ (the legal adult age is 19 in our study area). We also conducted the DD models for cardiovascular and respiratory mortality stratified by sex, age (20–64, 65+, 75+), and marital status. The differences in the estimated effectiveness of the heat wave warning system among strata were tested by the interaction test as suggested by Altman and Bland and as used in previous studies [34]. The statistical methods in our study were summarized in the flow chart of Supplementary Materials (Supplementary Figure S2).

## 3. Results

Table 2 shows characteristics of the mortality, heat wave, weather, and air pollution level data. The DD analysis included 50,002 all-cause deaths, 9917 cardiovascular deaths, and 4127 respiratory deaths. The number of all-cause mortalities during the eligible days for the analysis (50,002 deaths) was 25.7% of the total all-cause mortality for all days in the study period (194,409 deaths). The percentage of eligible days for DD analysis of the study regions (12,604 days) among all days in the study period (50,027 days) was 25.2%. The detection rate of the heat wave alert system for true heat wave (2202 days) or non-heat wave days (7,997 days) was 80.9% for the eligible days. The percentage of false negative heat wave days and false positive days for the eligible days was 6.1% and 13%, respectively. 

The percentages of false positive and negative heat wave days by year showed slightly increasing false positive heat wave days and decreasing false negative heat wave days since 2009 (Supplementary Table S2), implying a growing conservative approach of the alert system for detecting heat wave days. While Daegu is known as the hottest city in Korea, Daegu also showed the highest percentage of false negative heat wave days (3.85%) among the study cities. 

Figure 1 visualizes the concept of the computed average all-cause mortality (person/day) of each heat wave category for eligible days for analysis and the DD (∆) implying the effect of the heat wave warning system. The computed average all-cause mortality for this figure is not yet adjusted with population size of study region and baseline characteristics of weather and air pollution. The difference of mortality between true heat wave days (*E*[*Y*^1^(1)]) (4.072 deaths/day) and false positive heat wave days (*E*[*Y*^0^(1)]) (3.752 deaths/day) was smaller than the difference of mortality between false negative days (*E*[*Y*^1^(0)]) (4.435 deaths/day) and true non-heat wave days (*E*[*Y*^0^(0)]) (3.936 deaths/day), which indicates a reduction in mortality risk of heat waves attributable to the heat wave warning system.

**Figure 2:**
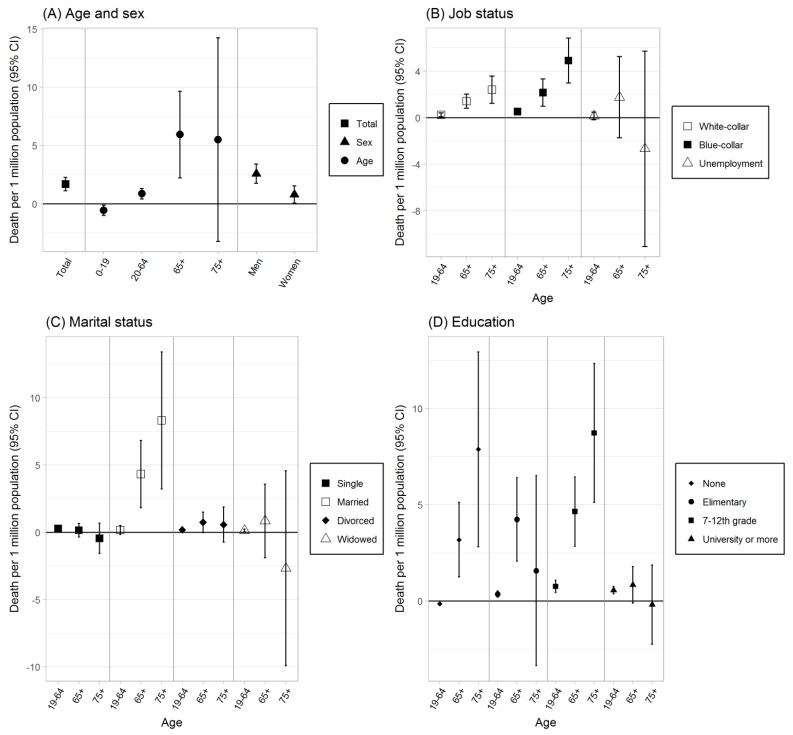
Effects of the heat wave warning system on all-cause mortality based on the comparison of mortality changes due to heat wave alert announcements for heat wave days versus non-heat wave days (i.e., counter factual) in 2009–2014 (mortality per 1,000,000 people per day). The effects are the estimates of difference-in-differences (DD) that were obtained by comparing mortality due to heat wave alert announcements for heat wave days versus mortality for non-heat wave days (i.e., counterfactual) through the difference-in-differences analysis. Negative estimates indicate reduction in mortality due to the heat wave alerts.

The estimated effects of the heat wave warning system on the risk of mortality of heat waves by population characteristics with adjustment of population size, weather, and air pollution are shown in Figure 2 and Supplementary Table S1. Despite the heat wave warning system, there was an increase in total all-cause mortality (1.687 per 1,000,000 population per day, [95% CI: 1.118, 2.255]) across all age groups for the study period (Figure 2A). Increases in mortality were found for all-cause mortality for the subpopulations of job status (Figure 2B) and marital status (Figure 2C). Evidence of effects of the heat wave warning system on reduction in mortality risk of heat waves was found for several subpopulations of age and education level: people aged 0–19 (−0.555 deaths/1,000,000 population per day, [95% CI: −0.993, −0.117]) (Figure 2A), and people aged between 19–64 who had no education (−0.114 deaths/1,000,000 population per day, [95% CI; −0.227, −0.061]) (Figure 2D). Based on the interaction test using the central estimates and their standard error with a significance level of 0.05 [34], the reductions in mortality in these subpopulations were statistically different from the effects of the heat wave warning system on mortality in the other age groups in each category (data not shown). 

To describe the sensitivity to weight truncation, the sensitivity analyses using propensity score weights truncated at the 99th percentile were shown in Supplementary Materials (Supplementary Table S3). Unlike the main result, under a less conservative setting with the truncation temperature, several additional subgroups showed significant reductions in all-cause mortality (indicating beneficial mortality effects of the heat wave warning system), including people aged 75+ who were unemployed and people aged 75+ who were widowed. 

The estimated effects of the heat wave warning system on cardiovascular and respiratory mortality by age, sex, job status, marital status, and education are shown in Table 3. Significant effects of reduced cardiovascular mortality from the heat wave warning system were found for people aged 75+ who were unemployed (−5.797 deaths/1,000,000 population per day [95% CI: −10.856, −0.739]), people aged 75+ who were widowed (−4.524 deaths/1,000,000 population per day [95% CI: −8.617, −0.431]), and people aged 19–64 with no education (−0.091 deaths/1,000,000 population per day [95% CI: −0.140, −0.042]). People aged 65+ who had university or higher degrees (−0.612 deaths per 1,000,000 population [95% CI: −1.140, −0.084]) and people aged 75+ who had education of university or higher degrees (−1.688 deaths/1,000,000 population [95% CI: −3.050, −0.325]) experienced a protective effect of the warning system. The reductions were statistically different from the estimates of the changes in mortality in the other age groups in each category according to the interaction test. Significant effects of reduced respiratory mortality were found for people aged 0-19 (−0.090 deaths/1,000,000 population per day [95% CI: −0.128, −0.053]), people aged 65+ who were widowed (−0.310 deaths/1,000,000 population per day [95% CI: −0.526, −0.094]), people aged 65+ who were single (−0.349 deaths/1,000,000 population per day [95% CI: −0.534, −0.163]), people aged between 19–64 who had no education (−0.032 deaths/1,000,000 population per day [95% CI: −0.055, −0.009]), people aged 65+ who had elementary education (−0.884 deaths/1,000,000 population per day [95% CI: −1.582, −0.185]), and people aged 75+ who had education of university or more degrees (−0.999 deaths/1,000,000 population per day [95% CI: −1.947, −0.050]). Except for the estimates for education attainment, differences in the estimated effects of the heat wave warning system among age-subgroups were significant for these reductions in respiratory mortality.

To illustrate potential changes over time, the DD analysis was applied to the two 3-year periods (2009–2011, 2012–2014) for the subgroups that showed significant effectiveness of the heat wave alerts on reducing mortality throughout the study period. The results (Table 4) showed decreases in mortality after the implementation that were attenuated to null over time for some groups such as people with elementary education (aged 65+ or 75+) and people who were widowed (age 65+ or 75+). On the other hand, people aged 19–64 with no education experienced delayed effects of the heat wave warning system on reducing mortality in the later period (2012–2014). Cardiovascular mortality for those who were widowed (aged 75+) also showed a delayed effect of the system. 

The sensitivity analysis applied to finer age subgroups in children found that the significant effects of the heat wave warning system on reduced all-cause or respiratory morality for children aged 0–19 were mainly due to the reduced mortality for children under age 5 (Supplementary Table S4). Due to small number of deaths, the DD estimates were not estimated for respiratory mortality in children aged 5–19. 

## 4. Discussion

Many countries have implemented nationwide or state- and local-level heat wave warning systems to minimize the risk of heat-related health problems [35]. While numerous epidemiologic studies focused on examining the relationship between high ambient temperature and mortality, few studies have reported changes in mortality risk during serial heat waves over years as a result of the increased awareness of the risk of heat in relation to the implementation of a heat wave warning system [36,37,38,39]. A study of Quebec, Canada applied a quasi-experimental method to evaluate the effectiveness of the Canadian heat action plans on mortality [40], finding reduced mortality attributable to the plans for elderly people and people living in low-education neighborhoods. A recent study examined if days with national heat wave alerts announced had significantly lower mortality risk compared to the comparable days but without alerts in 20 US cities using a case-crossover approach and found no significant association between the heat alerts and reduction in mortality [41]. To the best of our knowledge, our study is the first to assess the effectiveness of the national heat wave warning system and the subsequent governmental heat health plans on reducing mortality risk during heat waves by sub-groups of biological and socio-economic characteristics using a DD model combined with propensity score weighting approaches.

We found that the percentages of false negative and positive heat wave days (i.e., days with inaccurate heat wave alerts) varied by cities and years. Particularly, higher percentages of false negative and false positive heat wave days in Daegu, known as the hottest urban city in Korea, cast a substantial public health implication of heat-related health outcomes. In addition to the different exposure-response relationships for ambient temperature and mortality among regions, the variation of accuracy of the heat wave alerts may imply the need for regional-specific heat wave criteria.

Our results did not find a beneficial effect of the heat wave warning system for mortality of all-ages group in major 7 urban cities. These results may imply an incomplete achievement of public health benefits of the heat wave warnings and health plans, and suggest the opportunity to adjust the plans to maximize improvements to public health. However, we identified benefits for all-cause mortality in some subgroups. The negative DD estimates for all-cause mortality were coherently found in older persons (age 75+), which implies potential effectiveness of the heat wave warning system for reduced heat-related mortality for the target older age groups with vulnerable conditions (e.g., unemployment, being widowed), although the statistical power and the magnitude of the results were small. The additional significant estimates of DD for older subgroups under less conservative settings (i.e., by the 99th percentile truncation of the propensity scores) emphasize that our main results obtained in strictly conservative settings still claim significant decreases in mortality especially in older population. The positive DD estimates may indicate that mortality occurred beyond the level at which the intervention could function as a buffer preventing heat-related mortality. In the perspective of public health, the DD estimates for all-cause were constantly positive for younger old age group (age 65+) and young adults (aged under 65), which indicate that mortality risk from heat waves still remain for these subgroups in spite of implementation of the heat wave warning system.

The weak statistical power of the results for all-cause mortality in the elderly may be related with the relatively large fraction of deaths from tumor/neoplasm. According to the national statistics 2017 [42], the major cause of death in people aged 65 and above was tumor/neoplasm (26%) followed by cardiovascular diseases (24%), respiratory diseases (14%), and senility (6%). Our study suggested significant results for beneficial effects of the intervention component of the warning system on cardiovascular and respiratory mortality risk of heat waves in older persons (age 75+), particularly for those with additional susceptible factors such as unemployment, living alone, and low education attainment. In the context of vulnerability, living alone, having a lack of mobility and air-cooling system, and living on the top floor are considered to increase vulnerability to heat waves [43]. These factors are often correlated with age, which is also one of the main effect modifiers associated with vulnerability to heat-related mortality [44], while the evidence of sex as an effect modifier is less consistent [33,45]. The majority of heat wave health plans in many countries are aimed at the population over the age of 65 or 75 years and implement preventive measures for mitigating the health burdens within this vulnerable groups [46]. Targeting vulnerable groups including elderly population gives rise to a question on the difference in the changes in health impacts of heat waves across various age groups [44]. Our results indicate that elderly populations may have benefited from the heat wave health plans targeting this subpopulation. Also, our results support the previous study [47] of Korea that proposed a temporal decrease in heat-related mortality risk in elderly population (age 75+) after 2008 in which the national heat wave warning system was launched. As the age increases, prevalence of chronic diseases and activity limitations increase, resulting in differences in the health status between relatively younger and older elderly people. Given the rapid aging phenomenon in Korea in which more accessible health care for the elderly is demanded, our results showing the effects of heat wave warning system in the older elderly group (age 75+) are particularly interesting.

Furthermore, our results found that there was a temporal trend in the effectiveness of the heat wave warning system for those subgroups that experienced reduced mortality attributable to the heat wave alerts. A possible explanation for the trend is that people who immediately benefited from the heat wave warning system after its implementation were desensitized to the alerts and preventive measures. For the delayed effectiveness of the heat wave warning system for several groups, it is possible that a certain latent period existed for the warning system to be in effect. Given the ongoing reinforcement of the heat wave warning system, continuous examination in temporal trends of the effectiveness of the warning system intervention is crucial.

Due to differences in regional administrative resources and discretion for recommended governmental actions during heat wave days, the performance of the governmental heat wave health plans during heat wave days can vary by administrative regions [41]. Evaluation of regional performance of heat wave health plans is methodologically challenging. Although governmental officers may be concerned about the actual adherence of local offices to the national heat health plans, we did not have information on the performance of local offices for the national heat health plans and the presence of local specialized preventive actions for heat waves was not available. Future work could examine these issues to differentiate how specific implementations of heat wave warning systems affect health impacts. 

Behavioral changes after heat wave warnings may explain our findings of significant decreases in all-cause mortality in children aged 0–19 and young adults aged 19–64 with no education. An early access to prevention information such as leaflets may contribute to substantial individual behavioral changes, such as increased use of cooling systems and wet clothes in elderly people [48]. Voluntary actions performed by individuals who had access to the heat wave announcement from the media and were aware of preventive actions to reduce the health effects of heat waves could contribute to the reduced mortality during heat waves. Such actions include staying inside or under shade and drinking a sufficient amount of water. However, protective behaviors may not necessarily be due to heat wave announcements [20]; those who do not view themselves as vulnerable to heat may not take protective actions irrespective of actual vulnerability. In some cases, even people who consider themselves as vulnerable to heat may not change their behavior [41,49]. On the other hand, those who take voluntary preventive actions may do so not because of the heat wave alerts but due to their perception of hot weather [20]. Our analysis addresses this issue by distinguishing between heat wave days that are identified as those with heat wave warnings and those without warnings. Evidence on the awareness and tendency of taking preventive actions in population is not yet available in Korea; such information would allow more a stable investigation of the association between individual-level behaviors and improved health during heat waves, and thereby further inform which behaviors are most effective. 

In our study, significant evidence of the effectiveness of the heat wave warning system for reducing all-cause mortality related to heat waves was not found in white-collar nor blue-collar workers. Significant reduction in respiratory mortality was found in older blue-collar workers (i.e., aged 65+ or 75+). Numerous studies have reported significant impacts of heat waves or high temperature on mortality among workers such as farmers and labor workers at construction/extraction sites whose job is more likely related with performances at outdoors or hot environments [50,51]. Some previous work suggested that younger workers are more vulnerable to occupational injuries because they are likely to undertake more strenuous tasks, lack key training, and have not yet developed the same skills [52,53]. The prevention of heat-related illness and mortality at work sites can be improved by employer’s active support for occupational health preventive measures in addition to public health plans targeting the public. Preventive measures that are practical at work sites include monitoring the temperature and thermal stress indices, cooling systems, water intake management, shaded rest areas, and appropriate rest cycles and clothes [51,54]. The Ministry of Employment and Labor of Korea has announced the worker health prevention plans for heat waves, including the preventive measures mentioned above, and recommended that employers follow the plans since 2009 [55]. Further impact evaluation studies for the occupational health risks of heat stress would help develop evidence-based intervention programs targeting highly vulnerable occupations. 

Our results showed a significant reduction in cardiovascular and respiratory mortality attributable to the heat wave warning system in people aged 75+ years who were widowed. The effect of marital status was less robust to the selection of cut-offs for truncation of propensity score weights, likely due to small sample size. Generally, a married people may be less vulnerable as they could be cared for by their partners during hot periods. Evidence of the effect of marital status on the mortality risk of heat waves is relatively limited. Previous studies suggested an increased mortality risk in persons who are married or widowed/divorced during heat waves in Korea; São Paulo, Brazil; and northern China [56,57,58], whereas a study in Michigan showed higher risks of cardiovascular mortality during hot days in the unmarried elderly population [59]. A study in King County, Washington, US, did not find that marital status altered the mortality risk on hot days [60]. 

The main analysis results rely on the parallel trend assumption of the DD estimates. However, the changes in mortality during non-heat wave days may not be equal to the mortality change during heat wave days. The DD estimate can be positive even though there is in fact some reduction in heat-related mortality. A situation where the reduction in mortality during non-heat wave days is solely attractable to reduction in outdoor activities of the target population, which is an implausible assumption in reality but made for the discussion. In such a situation, we could consider a group of people who avoid outdoor activity during heat wave days regardless of the heat wave warning, but avoid it during non-heat wave days only when there is the warning. This group would have no effect of the warning during heat wave days, but some effect during non-heat wave days, which is a violation of the parallel trend assumption. For this subpopulation, since they will not participate in outdoor activities anyways during heat wave days, the warning system is not effective during heat wave days, but is effective during non-heat wave days. This violation can be severe if the proportion of this subpopulation is large but information on this proportion cannot be observed. Further research is required to estimate this proportion to check the plausibility of the parallel trend assumption. Further quantifications of the effectiveness of the intervention during the non-heat wave days with and without heat wave alerts (i.e., the counterfactual itself in our study setting) will be helpful to examine if the heat wave warning system plays a role as a preventive measure for days that are not extremely hot throughout warm seasons and contribute to reduction in mortality.

Although the statistically significant negative DD estimates coherently found in the elderly support the evidence of the beneficial mortality effects of the heat wave warning system for the elderly group, our results on the benefits of the heat wave warning system may necessitate cautious interpretation. The multiple testing problem (increases in the probability of wrongly rejecting a null hypothesis when performing multiple simultaneous hypothesis tests) [61] may have emerged as the DD method was applied to multiple different subpopulations. Nonetheless, the multiple testing problem was not prioritized over examining subgroup effects in our study, as our research aimed to examine how the effects of the heat wave warning system vary with the levels of vulnerability factors such as age or occupation. Multiple testing correction methods such as Bonferroni or Holms corrections give fewer insights on variation in vulnerability of subgroups. While the DD estimate is the specific linear combination of four quantities and the variance of it is the sum of each quantity’s variance, applying multiple testing corrections would result in very wide confidence intervals. Moreover, the results adjusted by multiple testing corrections would not provide informative insights on subgroup vulnerability as the DD estimates in our main results were relatively smaller compared to their large variance. We consider that our propensity score weighting method can provide reliable variance estimation while diminishing the potential confounding and selection biases for the effect estimates. With this context, our various subgroup analysis should not be considered as hypothesis generating research and finding hypotheses is not the main goal of this study. Future work examining the benefits of the heat wave warning system based on larger sample size and sophisticated statistical methods such as the bootstrap method would be useful to further verify the findings of this study. 

This study has several strengths. By combining temperature monitoring data and heat wave announcement data, we could determine the effectiveness of the national heat wave warning system for preventing mortality during heat waves. Our novel methods using the DD method combined with propensity score weighting enabled assessment of the changes in mortality that were attributable to the national heat wave warning system, with control for measured (e.g., air pollution) and unmeasured confounders. This provides convincing evidence to inform policy decisions [25]. Also, our methods, which are reasonable to apply to a warning system that is imperfect for detecting the days needing warnings, provide opportunities for evaluating heat-related public interventions, while only a few studies with limited geographical locations and populations have evaluated changes in heat-related health outcomes attributable to an administrative intervention using a quasi-experimental research design [40,62]. Another quasi-experimental design that can be feasible for the effect of heat wave warning system is the regression discontinuity model that can be used for intervention based on a continuous measure with a clearly defined threshold value for eligibility of enrollment to the intervention and the effectiveness of interventions in the vicinity of the threshold [25]. We note that this method was not applied to our study as our study question is if and how the heat wave warning system affects the mortality risk due to heat waves during the entire summer season in the whole population. Also, we reduced selection bias from incomparable comparison days to the hot enough days for heat wave warning announcement, by limiting the analysis to the identified eligible comparison days based on measured covariates. Another strength of this study is the investigation of impacts for subgroups of socio-economic status. 

Our study has some limitations. Our study is based on heat wave warnings, but does not address potential changes in individual behaviors that may occur in conjunction with heat wave warnings. We also did not have information of the ways in which the heat wave warnings were implemented in each region, which may have differences and reduce the comparability of changes between cities. We assumed that the monitoring stations within a given city represent the most accurate weather condition of the Gu, a smaller administrative unit than City. The same exposure level of all individuals within a city was assumed, using available monitoring data and heat wave announcement data; however, actual intra-city heat exposures may differ in an urban environment, especially due to the urban heat island effect. We focused on urban regions in an effort to have higher similarity in population for an appropriate study design; future work could provide comparisons of heat wave effects in urban areas versus rural areas. Lastly, we assumed that the heat wave alerts and subsequent governmental actions are activated according to the latest announcement of the heat wave alerts, for which the integrity (or level) of heat wave can be updated. Although this assumption was reasonable considering the update of information at the website of the Korea Meteorological Administration, it is not certain how urgently local officials receive the order for implementing heat wave health plans. Research that links heat wave warnings and implementation measures to health is needed to confirm these findings.

In summary, our study provides information on whether the heat wave warning system has contributed to a reduction in cause-specific mortality and which subpopulations benefited more from the heat wave warning system in Korea. The results indicated that several vulnerable subgroups experienced reduced all-cause mortality. The reduction in mortality might have been achieved through the heat actions plans implemented by local administrative officials and behavioral changes of individuals during days with an announced heat wave alert. Future study efforts are imperative to disentangle the effectiveness of these multiple pathways and find the most effective preventive measures for regional climate and local administrative capability. Continuous efforts on exploring changes in the effectiveness of the heat wave warning system over time are required to further support evidence-based policy making in relation to the health threats of climate change. 

## 5. Conclusions

We did not find an effect of heat wave alerts and health plans on the reduction in all-cause mortality for all ages in Korea. However, we found some evidence of effects of heat wave alerts and health plans for the reduction of mortality for several subpopulations based on characteristics such as age and socio-economic status. Further information on the various implementation responses by local governmental offices, adherence to these warnings, individual behavior patterns, and data for rural areas would help to provide additional information of the effectiveness of heat action plans that may vary by regions and individual vulnerability factors. 

## Figures and Tables

**Table 1:** Classification of heat wave (HW) days, risk assignments, and definition of Difference-in-Differences.

	Temperature Monitoring Data		Change
	Heat Wave Days	Non-Heat Wave Days	
HW alert +	*E*[*Y*^1^(1)]	*E*[*Y*^0^(1)]	*E*[*Y*^1^(1) − *Y*^0^(1))
HW alert −	*E*[*Y*^1^(0)]	*E*[*Y*^0^(0)]	*E*[*Y*^1^(0) − *Y*^0^(0)]
Difference	*E*[*Y*^1^(1) − *Y*^1^(0)]	*E*[*Y*^0^(1) − *Y*^0^(0)]	*Δ= E*[*Y*^1^(1) − *Y*^1^(0)] − *E*[*Y*^0^(1)*−Y*^0^(0)]

**Table 2:** Characteristics of mortality, heat wave, weather and air quality data (2009–2014).

Variable	All Days		Eligible Days * (26 °C < Daily Mean Temperature ≤ 30.5 °C)	
	Sum	(%)	Sum	(%)
Total	194,409	100	50,002	100
Sex				
Men	109,241	0.56	28,088	56.2
Women	85,168	0.44	21,914	43.8
Age				
Age 0–19	2711	1.4	715	1.4
Age 20–64	60,914	31.3	15,675	31.4
Age 65+	130,738	67.3	33,600	67.2
Cause-specific				
Cardiovascular disease	38,684	19.9	9917	19.8
Respiratory disease	16,491	8.5	4127	8.3
Job (People age ≥ 19)				
White-collar	13,574	7.0	3600	7.2
Blue-collar	22,597	11.6	5676	11.4
Unemployment*	151,520	77.9	38,970	77.9
Education (People age ≥ 19)				
None	30,622	15.8	8453	16.9
Elementary	52,077	26.8	13,468	26.9
7th–12th grade	47,046	24.2	12,456	24.9
University or more	56,562	29.1	13,366	26.7
Heat wave days				
True heat wave	2677	5.4	2202	17.5
True non-heat wave	44,576	89.1	7997	63.4
False positive heat wave	1956	3.9	1639	13.0
False negative heat wave	818	1.6	766	6.1
Monitored weather (mean, Q1–Q3)				
Daily maximum temperature (°C)	28.33	14.00–38.80	32.08	30.80–33.30
Daily mean temperature (°C)	23.97	22.00–33.20	27.62	26.70–28.40
Daily minimum temperature (°C)	20.56	18.40–29.40	24.12	23.10–25.20
Relative humidity (%)	73.59	65.60–82.10	71.57	65.40–78.60
Wind speed (m/s^2^)	2.06	1.30–2.50	2.18	1.40–2.70
Air quality (mean, Q1–Q3)				
O_3_ (ppm)	0.025	0.017–0.033	0.024	0.014–0.031
SO_2_ (ppm)	0.005	0.003–0.006	0.005	0.003–0.006
CO (ppm)	0.423	0.311–0.530	0.412	0.288–0.513
NO_2_ (ppm)	0.023	0.014–0.030	0.021	0.013–0.027
PM_10_ (µg/m^3^)	38.02	24.50–48.00	36.74	23.56–46.46

Notes. * Eligible days refers to days for which daily mean temperature were between 26.0 to 30.5 °C. The estimates represent values of any study region (Gu). Category ‘unemployment’ includes people who are unemployed, house-wives, and students.

**Table 3:** Effects of the heat wave warning system on changes in cardiovascular and respiratory mortality based on the comparison of mortality changes due to heat wave alert announcements for heat wave days versus non-heat wave days (i.e., counter factual) in 2009–2014 (mortality per 1,000,000 people per day).

	Cardiovascular Mortality		Respiratory Mortality	
	Estimate	(95% CI)	Estimate	(95% CI)
Age				
0–19	−0.016	(−0.072, 0.039)	−0.090	(−0.128, −0.053) *
20–64	0.261	(0.091, 0.432)	−0.014	(−0.082, 0.054)
65+	0.390	(−1.561, 2.341)	0.996	(−0.173, 2.165)
75+	0.266	(−4.523, 5.055)	2.838	(−0.067, 5.743)
Sex				
Men	0.605	(0.244, 0.965)	−0.102	(−0.330, 0.126)
Women	−0.065	(−0.444, 0.315)	0.326	(0.149, 0.503)
Job status (age)				
White-collar (19–64)	0.105	(0.056, 0.153)	−0.008	(−0.031, 0.015)
White-collar (65+)	0.099	(−0.261, 0.460)	0.379	(0.165, 0.593)
White-collar (75+)	−0.212	(−1.167, 0.744)	0.544	(0.010, 1.079)
Blue-collar (19–64)	0.150	(0.072, 0.228)	0.015	(−0.004, 0.035)
Blue-collar (65+)	−0.006	(−0.591, 0.578)	−0.598	(−0.961, −0.235) *
Blue-collar (75+)	−1.305	(−2.789, 0.179)	−0.748	(−1.480, −0.016) *
Unemployment (19–64)	0.108	(−0.011, 0.226)	0.040	(−0.007, 0.086)
Unemployment (65+)	−0.739	(−2.628, 1.149)	0.924	(−0.150, 1.997)
Unemployment (75+)	−5.797	(−10.856, −0.739) *	0.922	(−1.986, 3.831)
Marital status (age)				
Single (19–64)	0.030	(−0.030, 0.090)	0.028	(−0.001, 0.058)
Single (65+)	−0.044	(−0.374, 0.287)	−0.349	(−0.534, −0.163) *
Single (75+)	−0.409	(−1.285, 0.466)	−1.236	(−1.730, −0.742) *
Married (19–64)	0.185	(0.075, 0.294)	−0.015	(−0.057, 0.028)
Married (65+)	0.597	(−0.658, 1.853)	−0.301	(−1.090, 0.489)
Married (75+)	−1.294	(−4.523, 1.935)	−1.310	(−3.206, 0.585)
Divorced (19–64)	0.168	(0.092, 0.244)	−0.005	(−0.018, 0.008)
Divorced (65+)	0.057	(−0.383, 0.497)	1.292	(0.494, 2.090)
Divorced (75+)	0.061	(−1.013, 1.134)	3.090	(0.850, 5.330)
Widowed (19–64)	−0.025	(−0.058, 0.008)	0.020	(0.000, 0.041)
Widowed (65+)	−0.944	(−2.438, 0.550)	−0.310	(−0.526, −0.094) *
Widowed (75+)	−4.524	(−8.617, −0.431) *	−1.040	(−1.609, −0.470) *
Education (age)				
None (19–64)	−0.091	(−0.140, −0.042) *	−0.032	(−0.055, −0.009) *
None (65+)	−1.092	(−2.231, 0.047)	0.944	(0.341, 1.546)
None (75+)	−2.948	(−6.096, 0.199)	2.432	(0.764, 4.100)
Elementary (19–64)	0.090	(0.036, 0.145)	0.012	(−0.012, 0.036)
Elementary (65+)	0.631	(−0.597, 1.859)	−0.884	(−1.582, −0.185) *
Elementary (75+)	−1.669	(−4.888, 1.550)	−3.167	(−5.030, −1.305) *
7–12th grade (19–64)	0.323	(0.204, 0.441)	0.042	(−0.003, 0.088)
7–12th grade (65+)	0.860	(−0.116, 1.837)	0.647	(0.080, 1.214)
7–12th grade (75+)	0.260	(−2.226, 2.746)	2.617	(1.289, 3.944)
University or more (19–64)	0.040	(−0.014, 0.093)	0.005	(−0.003, 0.014)
University or more (65+)	−0.612	(−1.140, −0.084) *	−0.141	(−0.507, 0.226)
University or more (75+)	−1.688	(−3.050, −0.325) *	−1.566	(−2.518, −0.615) *

Notes. The effects are the estimates of difference-in-differences (DD) that were obtained by comparing mortality changes due to heat wave alert announcement for heat wave days versus that for non-heat wave days (i.e., counterfactual) through the difference-in-differences analysis. Negative estimates indicate reduction in mortality. Statistically significant results (significant level = 0.05) are marked with an asterisk (*).

**Table 4:** Trend in the effects of the heat wave warning system on changes in cardiovascular and respiratory mortality per 1,000,000 people per day.

	Study Period (2009–2014)		Period 1 (2009–2011)		Period 2 (2012–2014)	
	Estimate	(95% CI)	Estimate	(95% CI)	Estimate	(95% CI)
All-cause mortality						
Marital status (age)						
Widowed (65+)	0.844	(−1.895, 3.583)	−8.919	(−13.533, −4.306) *	2.795	(−1.296, 6.887)
Widowed (75+)	−2.671	(−9.906, 4.563)	−21.640	(−35.97, −7.310) *	−3.673	(−15.721, 8.376)
Education (age)						
None (19–64)	−0.144	(−0.227, −0.061) *	−0.065	(−0.188, 0.057)	−0.401	(−0.544, −0.258) *
Elementary (65+)	4.239	(2.070, 6.408)	−6.979	(−10.693, −3.266) *	3.995	(0.466, 7.524)
Elementary (75+)	1.577	(−3.359, 6.512)	−18.660	(−28.563, −8.757) *	−0.004	(−9.234, 9.226)
Cardiovascular mortality						
Marital status (age)						
Widowed (65+)	−0.944	(−2.438, 0.550)	−0.880	(−3.396, 1.636)	−1.409	(−3.604, 0.787)
Widowed (75+)	−4.524	(−8.617, −0.431) *	−2.471	(−10.24, 5.298)	−9.140	(−15.776, −2.504) *
Education (age)						
None (19–64)	−0.091	(−0.140, −0.042) *	−0.005	(−0.053, 0.043)	−0.284	(−0.382, −0.187) *
Elementary (65+)	0.631	(−0.597, 1.859)	0.100	(−1.784, 1.984)	0.510	(−1.352, 2.372)
Elementary (75+)	−1.669	(−4.888, 1.550)	−1.911	(−6.938, 3.116)	−1.650	(−6.956, 3.657)
Respiratory mortality						
Marital status (age)						
Widowed (65+)	−0.944	(−2.438, 0.550)	0.714	(−0.594, 2.021)	2.158	(0.894, 3.423)
Widowed (75+)	−4.524	(−8.617, −0.431) *	1.239	(−2.840, 5.318)	6.705	(2.778, 10.632)
Education (age)						
None (19–64)	−0.032	(−0.055, −0.009) *	0.008	(−0.013, 0.030)	−0.167	(−0.221, −0.114) *
Elementary (65+)	−0.884	(−1.582, −0.185) *	−2.024	(−3.084, −0.964) *	2.084	(1.025, 3.142)
Elementary (75+)	−3.167	(−5.030, −1.305) *	−5.123	(−8.204, −2.041) *	5.752	(2.686, 8.818)

Notes. The effects are the estimates of difference-in-differences (DD) that were obtained by comparing mortality changes due to heat wave alert announcement for heat wave days versus that for non-heat wave days (i.e., counterfactual) through the difference-in-differences analysis. Negative estimates indicate reduction in mortality. Statistically significant results (significant level = 0.05) are marked with an asterisk (*).

## Supplementary Materials

The following are available online at https://www.mdpi.com/1660-4601/16/12/2245/s1, Figure S1: Changes in proportions (%) of the types of heat wave days for intervals of daily mean temperature (°C), Figure S2: Flow chart of the statistical methods, Table S1: Effects of the heat wave warning system on all-cause mortality per 1,000,000 people per day (2009–2014), Table S2: Percentages of false positive and false negative heat wave days by city and year, Table S3: Estimated effects of the heat wave warning system on cardiovascular and respiratory deaths per 1,000,000 population per day, based on propensity score weights truncated at the 99 percentile, Table S4: Effects of effects of the heat wave warning system on mortality in different age groups of children and adolescents (2009–2014).
