# Supplementary material for: The Use of a Quasi-Experimental Study on the Mortality Effect of a Heat Wave Warning System in Korea
Source: Int J Environ Res Public Health. 2019 Jun 25;16(12):2245. doi: 10.3390/ijerph16122245 (PMC6617315; doi:10.3390/ijerph16122245)
Supplement: Supplementary file 1 [file ijerph-16-02245-s001.pdf]

International Journal of
Environmental Research and Public Health

Supplementary Materials: The Use of a Quasi-Experimental Study on the Mortality Effect of a Heat Wave Warning System in South Korea

Seulkee Heo [1,*], Amruta Nori-Sarma [1], Kwonsang Lee [2], Tarik Benmarhnia [3], Francesca Dominici [2] and Michelle L. Bell [1]

[1] School of Forestry and Environmental Studies, Yale University, New Haven, CT 06520, USA; amrutasri.nori-sarma@yale.edu (A.N.-S.); michelle.bell@yale.edu (M.L.B.)
[2] Harvard T. H. Chan School of Public Health, Harvard University, Boston, MA 02115, USA; kwonsanglee@hsph.harvard.edu (K.L.); fdominic@hsph.harvard.edu (F.D.)
[3] Department of Family Medicine and Public Health and Scripps Institution of Oceanography, University of California San Diego, La Jolla, CA 92093, USA; tbenmarhnia@ucsd.edu
* Correspondence: seulkee.heo@yale.edu

Contents.

Figure S1. Changes in proportions (%) of the types of heat wave days for intervals of daily mean temperature (°C).

Figure S2. Flow chart of the statistical methods.

Table S1. Effects of the heat wave warning system on all-cause mortality per 1,000,000 people per day (2009-2014).

Table S2. Percentages of false positive and false negative heat wave days by city and year.

Table S3. Estimated effects of the heat wave warning system on cardiovascular and respiratory deaths per 1,000,000 population per day, based on propensity score weights truncated at the 99 percentile.

Table S4. Effects of effects of the heat wave warning system on mortality in different age groups of children and adolescents (2009-2014).

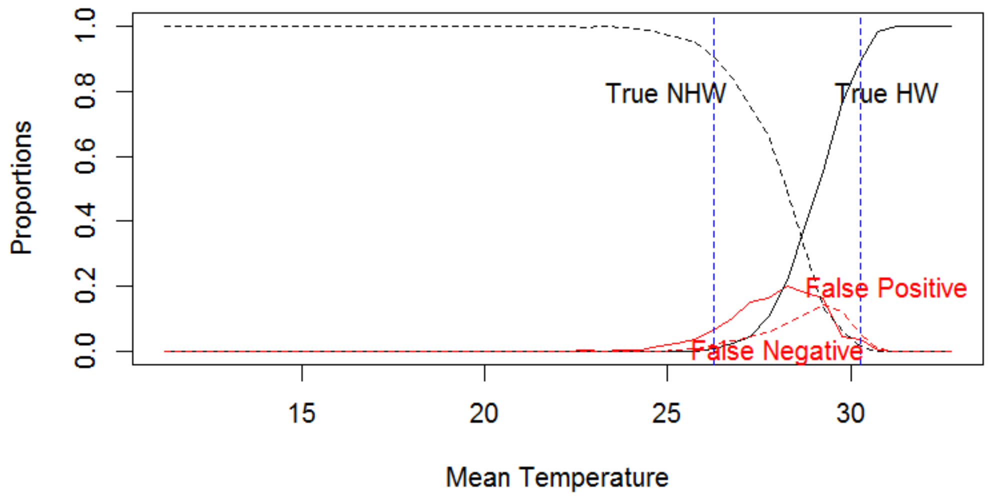

Figure S1. Changes in proportions (%) of the types of heat wave days for intervals of daily mean temperature (°C).

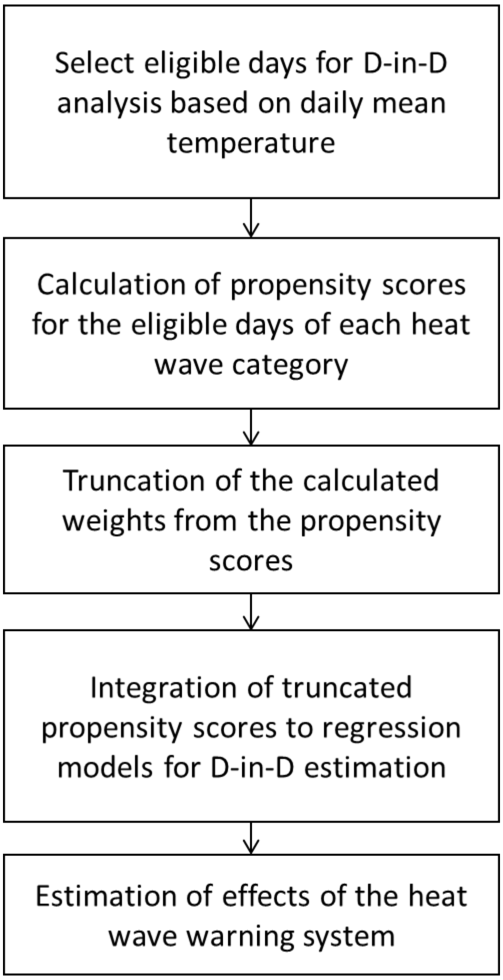

Figure S2. Flow chart of the statistical methods.

International Journal of Environmental Research and Public Health

MDPI

Table S1. Effects of the heat wave warning system on all-cause mortality per 1,000,000 people per day (2009–2014).

	Estimate	(95% CI)
Total	1.687	(1.118, 2.255)
Sex		
Men	2.579	(1.749, 3.409)
Women	0.795	(0.054, 1.537)
Age		
0–19	−0.555	(−0.993, −0.117) *
20–64	0.858	(0.412, 1.324)
65+	5.939	(2.215, 9.663)
75+	5.500	(−0.236, 14.237)
Job status (age)		
White-collar (19–64)	0.249	(0.079, 0.418)
White-collar (65+)	1.423	(0.812, 2.033)
White-collar (75+)	2.400	(1.233, 3.567)
Blue-collar (19–64)	0.520	(0.291, 0.750)
Blue-collar (65+)	2.166	(1.001, 3.330)
Blue-collar (75+)	4.909	(2.976, 6.841)
Unemployment (19–64)	0.147	(−0.19, 0.484)
Unemployment (65+)	1.764	(−1.736, 5.263)
Unemployment (75+)	−2.673	(−11.068, 5.722)
Marital status (age)		
Single (19–64)	0.294	(0.088, 0.501)
Single (65+)	0.164	(−0.336, 0.663)
Single (75+)	−0.440	(−1.568, 0.688)
Married (19–64)	0.186	(−0.147, 0.519)
Married (65+)	4.323	(1.816, 6.831)
Married (75+)	8.315	(3.222, 13.409)
Divorced (19–64)	0.201	(0.009, 0.393)
Divorced (65+)	0.741	(−0.014, 1.496)
Divorced (75+)	0.577	(−0.718, 1.872)
Widowed (19–64)	0.154	(0.057, 0.251)
Widowed (65+)	0.844	(−1.895, 3.583)
Widowed (75+)	−2.671	(−9.906, 4.563)
Education (age)		
None (19–64)	−0.144	(−0.227, −0.061) *
None (65+)	3.187	(1.253, 5.120)
None (75+)	7.886	(2.823, 12.949)
Elementary (19–64)	0.363	(0.205, 0.521)
Elementary (65+)	4.239	(2.070, 6.408)
Elementary (75+)	1.577	(−3.359, 6.512)
7–12th grade (19–64)	0.764	(0.446, 1.081)
7–12th grade (65+)	4.646	(2.845, 6.447)
7–12th grade (75+)	8.733	(5.127, 12.34)
University or more (19–64)	0.564	(0.372, 0.756)
University or more (65+)	0.845	(−0.101, 1.792)
University or more (75+)	−0.189	(−2.250, 1.872)

Notes. Statistically significant results (significant level = 0.05) are marked with an asterisk (*).

Table S2. Percentages of false positive and false negative heat wave days by city and year.

	False Positive Heat Wave Days	False Negative Heat Wave Days		False Positive Heat Wave Days	False Negative Heat Wave Days
City	Percent (%)	Percent (%)	Year	Percent (%)	Percent (%)
Seoul	3.2%	1.6%	2009	1.4%	1.4%
Busan	3.5%	0.8%	2010	5.1%	2.6%
Daegu	6.5%	3.8%	2011	2.7%	2.0%

Incheon	0.5%	3.0%	2012	5.0%	1.2%
Gwangju	6.3%	0.1%	2013	7.7%	1.8%
Daejeon	5.0%	1.0%	2014	1.6%	1.0%
Ulsan	7.5%	0.8%			

Table S3. Estimated effects of the heat wave warning system on cardiovascular and respiratory deaths per 1,000,000 population per day, based on propensity score weights truncated at the 99 percentile (2009–2014).

	**Estimate (95% CI)**	
Total	1.176	(0.596, 1.757)
Age		
0–19	0.266	(−0.101, 0.634)
20–64	0.503	(0.036, 0.969)
65+	1.567	(−2.221, 5.354)
75+	−6.082	(−14.973, 2.808)
Sex		
Men	2.249	(1.401, 3.098)
Women	0.099	(−0.653, 0.851)
Job status (age)		
White-collar (19–64)	0.047	(−0.127, 0.222)
White-collar (65+)	0.812	(0.181, 1.442)
White-collar (75+)	2.076	(0.851, 3.300)
Blue-collar (19–64)	0.407	(0.173, 0.640)
Blue-collar (65+)	3.178	(2.012, 4.345)
Blue-collar (75+)	5.350	(3.339, 7.362)
Unemployment (19–64)	0.088	(−0.257, 0.434)
Unemployment (65+)	−2.833	(−6.388, 0.723)
Unemployment (75+)	−14.144	(−22.669, −5.618) *
Education (age)		
None (19–64)	0.006	(−0.064, 0.076)
None (65+)	1.941	(0.027, 3.855)
None (75+)	5.793	(0.802, 10.783)
Elementary (19–64)	0.420	(0.256, 0.584)
Elementary (65+)	5.339	(3.128, 7.551)
Elementary (75+)	4.256	(−0.756, 9.267)
7–12th grade (19–64)	0.571	(0.242, 0.900)
7–12th grade (65+)	4.652	(2.816, 6.487)
7–12th grade (75+)	7.468	(3.75, 11.185)
University or more (19–64)	0.410	(0.213, 0.607)
University or more (65+)	0.277	(−0.704, 1.258)
University or more (75+)	−1.325	(−3.473, 0.823)
Marital status (age)		
Single (19–64)	0.150	(−0.057, 0.356)
Single (65+)	0.063	(−0.457, 0.583)
Single (75+)	−0.707	(−1.880, 0.467)
Married (19–64)	0.037	(−0.305, 0.380)
Married (65+)	3.102	(0.543, 5.660)
Married (75+)	6.456	(1.223, 11.688)
Divorced (19–64)	0.103	(−0.093, 0.298)
Divorced (65+)	0.401	(−0.382, 1.183)
Divorced (75+)	0.550	(−0.795, 1.896)
Widowed (19–64)	0.179	(0.079, 0.280)
Widowed (65+)	−1.837	(−4.616, 0.942)
Widowed (75+)	−12.051	(−19.373, −4.730) *

Notes. Statistically significant results (significant level = 0.05) are marked with an asterisk (*).

Table S4. Effects of effects of the **heat** wave warning system on mortality in different age groups of children and adolescents (2009–2014).

	Estimate	(95% CI)
All-cause		
Age 0–4	−4.570	(−6.305, −2.835) *
Age 5–19	0.480	(0.151, 0.809)
Age 0–19	−0.555	(−0.993, −0.117) *
Cardiovascular		
Age 0–4	0.027	(−0.035, 0.090)
Age 5–19	−0.025	(−0.092, 0.041)
Age 0–19	−0.016	(−0.072, 0.039)
Respiratory		
Age 0–4	−0.436	(−0.615, −0.258) *
Age 5–19	NA	
Age 0–19	−0.090	(−0.128, −0.053) *

Notes. Due to small number of deaths, the DD estimates were not estimated for respiratory mortality in children aged 5–19.
